# Foraging Ecology Predicts Learning Performance in Insectivorous Bats

**DOI:** 10.1371/journal.pone.0064823

**Published:** 2013-06-05

**Authors:** Theresa M. A. Clarin, Ireneusz Ruczyński, Rachel A. Page, Björn M. Siemers

**Affiliations:** 1 Sensory Ecology Group, Max Planck Institute for Ornithology, Seewiesen, Germany; 2 Mammal Research Institute PAS, Białowieża, Poland; 3 Smithonian Tropical Research Institute, Panama, Panama; Università degli Studi di Napoli Federico II via Università, Italy

## Abstract

Bats are unusual among mammals in showing great ecological diversity even among closely related species and are thus well suited for studies of adaptation to the ecological background. Here we investigate whether behavioral flexibility and simple- and complex-rule learning performance can be predicted by foraging ecology. We predict faster learning and higher flexibility in animals hunting in more complex, variable environments than in animals hunting in more simple, stable environments. To test this hypothesis, we studied three closely related insectivorous European bat species of the genus *Myotis* that belong to three different functional groups based on foraging habitats: *M. capaccinii*, an open water forager, *M. myotis*, a passive listening gleaner, and *M. emarginatus*, a clutter specialist. We predicted that *M. capaccinii* would show the least flexibility and slowest learning reflecting its relatively unstructured foraging habitat and the stereotypy of its natural foraging behavior, while the other two species would show greater flexibility and more rapid learning reflecting the complexity of their natural foraging tasks. We used a purposefully unnatural and thus species-fair crawling maze to test simple- and complex-rule learning, flexibility and re-learning performance. We found that *M. capaccinii* learned a simple rule as fast as the other species, but was slower in complex rule learning and was less flexible in response to changes in reward location. We found no differences in re-learning ability among species. Our results corroborate the hypothesis that animals’ cognitive skills reflect the demands of their ecological niche.

## Introduction

Ecological demands have been postulated as a driving factor in the evolution of cognitive complexity and intelligence [1], [2]. The cognitive abilities of animals are often well adapted to the requirements of their ecological niche (e.g. [3], [4]). Migratory bird species, for example, have much longer long-term memory than non-migratory species, and food-storing bird species show better spatial memory than non-storing species [5], [6]. The ecological context of feeding affects learning abilities in crabs: mobile species show experience-dependent modifications of foraging behavior while sedentary species do not [7]. In lizards, congeneric species with different foraging strategies display the same learning abilities but actively foraging species performed better in a reversal visual discrimination task than sit-and-wait predators [8]. The spatial learning abilities of voles reflect the complexity of their foraging habitats and their dietary specializations, with slower learning and decreased flexibility in more specialized species that forage in less complex habitats [9]. Game theory modeling also indicates that the unpredictability of food resources increases social foraging as well as generalism in diet; factors which can shape the evolution of cognition [1]. According to the “Environmental Complexity Thesis” [10], the heterogeneity of an environment in space and time is thought to be one of the key factors that determine the rate of the evolution of cognitive skills [1], [10], [11]. In our study, we compare learning and flexibility in insectivorous bats to investigate the influence of an animal’s ecological niche – specifically the complexity of its foraging habitat and the degree of stereotypy in its foraging behavior – on its cognitive abilities.

Bats are especially well-suited for investigations of ecological adaptations. Following rodents they are the second most species rich mammalian order [12] and show great ecological diversity. Their diet ranges from nectar, pollen, and fruit to insects, small vertebrates, and blood [13]. We find high ecological diversity even among closely related species, and similar ecologies have developed convergently in many distantly related groups. Despite phylogenetic distance, similar wing morphology [14] and echolocation patterns (e.g. [15]–[18]) have emerged in bats foraging in similar habitat types. *Macrophyllum macrophyllum*, for instance, is the only phyllostomid bat that forages exclusively over water [19]. It uses distinct terminal groups of echolocation calls prior to catching its prey, an echolocation behavior that is unique among phyllostomid bats but similar to distantly related trawling bat species [20], [21] and is thus clearly shaped by the specieś foraging behavior rather than its phylogeny [18].

The similarities in morphology and behavior among distantly related but ecologically similar bats make it reasonable to expect that the cognitive abilities of bats are also shaped by the demands of their respective niche [22] and that closely related but ecologically divergent species will differ in their abilities to solve cognitive tasks. In bats, it has been shown that wing size, which reflects foraging habitat density, is correlated to larger hippocampi, which are known to relate to better spatial memory in a wide range of animal taxa [23]. We investigated whether learning performance and behavioral flexibility vary with foraging ecology by comparing closely related species that differ in their foraging behavior. Can one predict the learning performance of a species from the complexity of the habitat in which it forages? We hypothesize that species hunting in structurally complex habitats that fluctuate over time should learn faster and should be more flexible than species hunting in less complex, more stable habitats.

Insectivorous bats can be categorized into different functional groups according to habitat complexity, temporal habitat stability, and the sensory basis of prey detection. In a simplified overview, three groups can be distinguished: bats that use echolocation to forage in the open, either in open air or over water; bats that use prey-emitted acoustic cues to glean from vegetation or open ground; and bats that use echolocation to hunt prey near, but not on, vegetation (for a detailed review see [17]). For our study, we chose one representative from each group, each from the genus *Myotis*. We experimentally tested flexibility and learning performance in these species of closely-related, congeneric European bat species to test the hypothesis that foraging ecology predicts cognitive ability.

Water foraging bats use echolocation to hunt insects over water surfaces (open water foragers). Their foraging task should be the least demanding within the three groups because the echoes reflected from insects are not masked by echoes reflected from background structures. A water surface acts as an acoustic mirror reflecting almost all the sound energy away from the bats and returning only the echoes of the bat’s prey [24]–[27]. Bodies of water tend to be uniform and unstructured and do not undergo large changes in an observable time span; they usually do not change from night to night. Insect abundance over water surfaces also does not seem to alter the stereotyped behavior of water foraging bats: analysis of hunting behavior of the open water foraging bat, *Myotis daubentonii*, under natural conditions showed no effect of food abundance on flight activity. It is possible that the high degree of stereotypy in behavior in this group of bats is due to uniformly high insect abundance in water foraging habitats [28], [29]. We predict that associative learning between abundance of insect prey and specific locations or shapes plays little role in the hunting behavior of water foraging bats. As a representative of water foraging bats, we chose *M. capaccinii,* a species found in the Mediterranean region that hunts over water surfaces, with a preference for slow running rivers [30], [31].

Bats from the second group glean arthropods from open ground or vegetation. In this situation insect echoes are masked by strong background-generated clutter echoes. Gleaning bats are specialized to find their prey by listening for prey-generated sounds such as rustling noises or communication sounds [32] and are therefore termed “passive listeners”. Their foraging habitats are highly structured and undergo large changes over the course of a year. Consequently, these bats must be adept at learning to recognize specific landscape features, such as a freshly cut meadow, as a good foraging ground (e.g. [33]). They could also learn to associate prey profitability with specific sensory cues, such as rustling noise amplitude [34]. We predict that associative learning should play a more important role in the foraging behavior of passive listeners than open water foragers. To represent the passive listening gleaners, we chose *M. myotis*, a species which hunts arthropods from open accessible ground in forests or fields [35]–[37].

The third group, termed clutter specialists, consists of bats using echolocation to forage for insects in close proximity to vegetation. This foraging task is extremely challenging as it requires the ability to distinguish clutter echoes from the echoes of insects (e.g. [38], [39]). Like passive listening gleaners, the foraging environment of clutter specialists changes rapidly: small plants and flowers appear and disappear, while trees grow leaves, blossoms and fruits and rapidly loose them again. Most important for bats, insect abundance changes in space and time as a function of plant phenology (e.g. [40]). Because flowering plants attract insects, the bats likely have to constantly build and rebuild associations with certain plants and places that are linked to high prey abundance [22]. Associative learning of cues that indirectly indicate the presence of prey, and the flexibility to update these associations rapidly over time, should thus be more important in these bats than in the former two groups. As a representative of this group, we investigated *M. emarginatus*, a clutter specialist sympatric with the other two species in our study [41]–[44].

**Figure 1:**
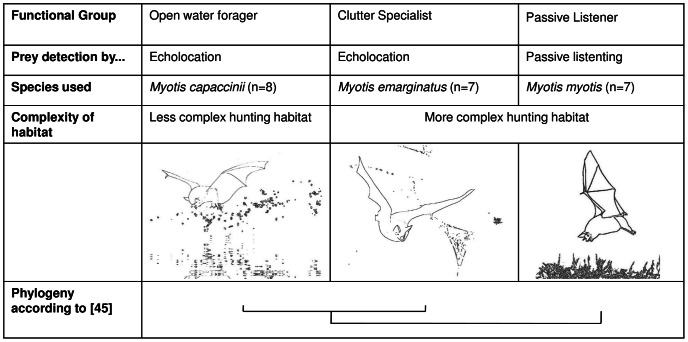
The bat species used in the experiments were closely related European congeners representing three distinct foraging guilds.

We specifically chose three closely related species (illustrated in Figure 1) to decrease the likelihood that species differences could be attributed to phylogenetic distance. Our species choice is conservative in that *M. myotis* and *M. emarginatus* are more similar in their foraging ecology, but more distantly related to each other than each is to the ecologically dissimilar *M. capaccinii* [45].

Based on the hypothesis that the complexity of the foraging habitat predicts cognitive ability, we expected open water foragers to display relatively stereotyped behavior, slow learning and low flexibility. In contrast, we predicted that passive listening gleaners and clutter specialists would be fast learners and highly flexible [22]. To compare learning performance and flexibility, we used a purposefully artificial and thus species-fair plastic maze in which the animals had to crawl and search for food. A similar crawling paradigm has been successfully used for bats in other simple learning tests [46], [47]. We quantified the bats’ behavior on four tasks: exploration, simple rule learning, a reversal learning task that tested for flexibility, and complex rule learning. We predicted that the two species hunting in or near vegetation would be faster learners and show greater flexibility than the water foraging species.

## Materials and Methods

### Ethics Statement

Capture and husbandry were conducted in accordance with the species-specific recommendations of the Canadian Council on Animal Care on bats [48] and were licensed by the responsible Bulgarian authorities (MOEWSofia and RIOSV-Ruse, permit numbers 193/01.04.2009 and 205/29.05.2009). Officials from the Bulgarian Ministry of Environment and Water (MOEW) inspected our work in accordance with Section 8, Article 23, Paragraph 3 and 4 of the Bulgarian Biodiversity Law. According to Bulgarian laws no further ethical approval by a committee is required for a non-invasive behavioral study. No bats were harmed. All bats were released in good health, at or above capture weight, at their respective capture sites after the experiments.

### Animals

Bat capture and experiments were conducted in Bulgaria. We used experimentally naïve, wild-caught adult male bats of the species *M. myotis* (n = 7), *M. capaccinii* (n = 8) and *M. emarginatus* (n = 7). We captured *M. myotis* and *M. capaccinii* in or near the entrance of caves in northeastern Bulgaria. *M. emarginatus* were mist-netted in the central Balkan Mountains near Gabrovo. The animals were then transferred to the Tabachka Bat Research Station (Bulgaria) of the Sensory Ecology Group (Max Planck Institute for Ornithology, Seewiesen, Germany), which is run in cooperation with the directorate of the Rusenski Lom Nature Park in the district of Ruse. *M. capaccinii* (7–10 g) and *M. emarginatus* (6–9 g) were housed together in a screen tent (2.2 m×0.9 m×1.1 m). *M. myotis* (20–27 g) were kept in a holding cage (50 cm×35 cm×40 cm). All animals had ad libitum access to water. For individual recognition, we gave all bats a within-species individual-specific haircut by cutting a small stripe of hair on one part of the back. After capture, the bats were hand-fed live mealworms (*Tenebrio molitor*) for two nights before starting training in the maze, to allow them time to adjust to the environment and the new food source. *M. myotis* received 4 g of mealworms per day, while *M. capaccinii* and *M. emarginatus* received 1.5 g and 1.7 g respectively. All bats readily accepted mealworms as food. When experiments began, bats were only fed in the experiment (with the exception of night 1, see below). The body mass of the bats was measured before and after each session to ensure that the animals maintained their weight. The dark-light-cycle and temperature in captivity mirrored ambient, natural conditions. The experiments with *M. capaccinii*, *M. myotis*, and four *M. emarginatus* were conducted in July and the first half of August. The experiments with the other three *M. emarginatus* took place in September of the same year. After the experiments all bats were released at their respective capture sites.

### Experimental Setup

**Figure 2:**
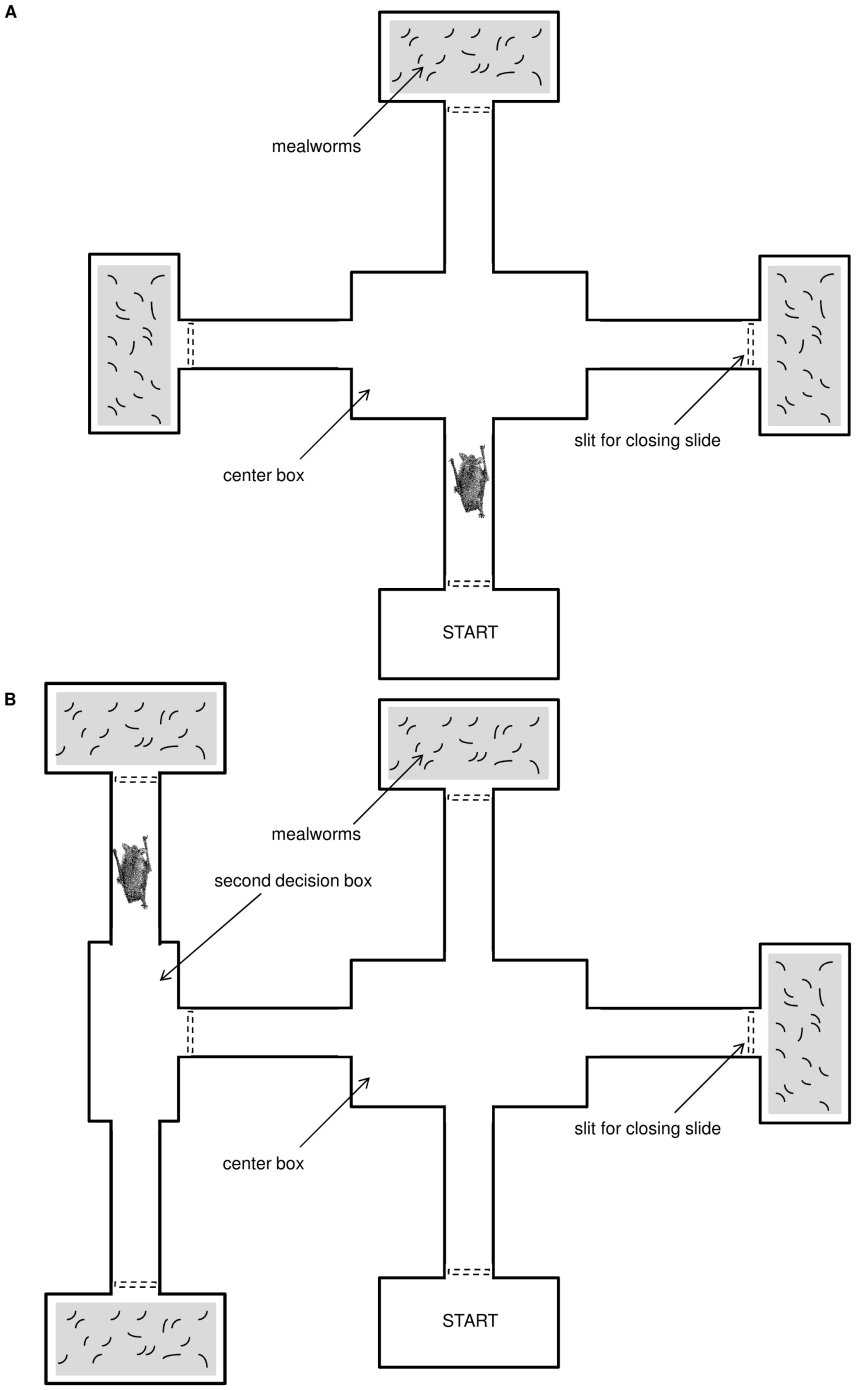
Mazes used in experiments. a) Maze used in experiments 1 to 3; “Exploration”, “Simple Rule Learning”, “Flexibility and Re-learning” b) Extended Maze used in experiment 4; “Complex Rule Learning”.

Experiments were conducted in a plastic maze. The simple form consisted of 4 plastic boxes (20 cm×13.5 cm×10 cm) connected to a large center box (24 cm×16.5 cm×12.5 cm) by plastic tubes (25 cm long, 7 cm internal diameter). All bats could easily crawl and turn in the tubes. One of the four boxes always served as the starting box while the other three contained mealworms (see Figure 2a). In all experiments, we placed the same amount of live mealworms in each feeding box to prevent the bats from receiving different acoustic, visual or olfactory cues from the three boxes. *M. myotis* was presented with normal-sized mealworms (approx. 0.1 g each) while the two smaller species received small mealworms (approx. 0.05 g each). The mealworms could crawl in the feeding boxes, but could not move back into the plastic tubes, as the entrance to the tubes was elevated by about 0.5 cm. The entrance to each box could be closed with a plastic slide. Additional arms could be added to the maze for the complex task (see Figure 2b). To remove possible olfactory cues, the mazes were cleaned after each experimental session with detergent and water and a clean set-up was used for each bat. Experiments were conducted in near darkness. The only light source was dim red light from the observer’s headlamp (Tactikka plus, PETZL) and an infrared light (CONRAD, 1/3″ CMOS colour camera with IR) mounted on the ceiling to enable video recording. A camera (Watec, WAT-902H2 Ultimate) was placed above the maze to record all experiments. The videos were recorded on miniDV tapes with a camcorder (Sony DCR-TRV80E recorder).

### Procedure

#### Experiment 1: Exploration

On night 1 we conducted an exploration experiment. The experiment began when a bat was placed into the maze and left there for 15 minutes. Prior to placing the bat in the maze, each box, including the center box, was baited with three mealworms. The bats were allowed to move freely and eat as many of the mealworms as they wanted to. Thus, the bats had the possibility to acclimate to the plastic maze and we were able to use video analysis of movement in the maze to assess the possible effect of species differences in body size and crawling performance on the results of the following experiments. After 15 minutes of exploration, the bat was removed from the maze and was additionally hand-fed up to the normal daily amount of food taking into account the number of mealworms eaten during exploration.

We analyzed the videos of the exploration experiment for several behavioral parameters to control for differences in crawling abilities. “Small scale exploration” was defined as the amount of time an animal spent exploring one of the boxes or exploring an arm systematically by moving back and forth. In contrast “fast walking” was defined as the amount of time an animal was crawling straight through the maze; and “immobile” referred to times when the animal was not moving. We also quantified the “number of boxes visited” (excluding the start box), the “number of mealworms eaten”, and the “latency to exit the start box”. We additionally examined whether an animal explored the center box thoroughly or only crossed it when crawling from one arm to another. Two videos from *M. capaccinii* were lost due to technical failure. We analyzed the data using Anova, Kruskal-Wallis-test and Chi-square-test where appropriate.

#### Training

During the next two nights, the bats were trained to find rewards in one of the boxes (half of the bats were trained to the right box; half of the bats to the left box; balanced within species, fig. 2a). To train the bats to feed from the rewarded box, the other two arms were blocked by slides. In this training phase and all following experiments, each night, one session of 10 trials (one trial being one searching event) was conducted for each animal. At the beginning of each trial the bat was placed into the starting box. When it reached the target box it was allowed to eat two to four mealworms. Afterwards it was removed from the target box and the next trial started. If a bat did not move for more than five minutes (either did not leave the starting box or stopped in the center box), the trial was aborted. Then, after a break of two to five minutes, the next trial started.

#### Experiments 2–4

We conducted three additional experiments with all bats. All experiments were conducted in the same order. Each bat was tested in each experiment for at least three sessions (one session per night). Within each session after trial 4 and 7 the arms of the maze were interchanged to prevent the bat from following its own scent. After trial 7 the center box was rotated 180°.

If a bat made 8 out of 10 correct decisions, it was considered to have learned the task. To give the animals the opportunity to better consolidate the newly acquired information, individuals were required to make 8 out of 10 correct decisions on two consecutive nights before moving on to the next experiment. If a bat did not learn a task after 15 sessions, it was removed from the experiment. This rule only had to be applied for one *M. capaccinii*. The whole study required each bat to stay in captivity for a minimum of 14 nights (two nights of hand-feeding, one night in experiment 1, two nights training and three nights for each of the experiments 2–4). Theoretically, the study could take as long as 50 nights (2+1+2+15+15+15) for one individual, although no bat required the maximum amount of time in the experiments.

#### Experiment 2: Simple rule learning

On night 4 the second experiment started. For this and all subsequent experiments, all four arms of the maze were open. After reaching the center box, the bat was required to enter the arm in which it had formerly been trained to find mealworms (fig. 2a). In the target box it was again allowed to eat two to four mealworms before the next trial started. If a bat entered an unrewarded arm, the entrance to the box was blocked with a plastic slide. Subsequently, the bat was removed from the maze without receiving a food reward and the next trial started. We scored the number of days that an animal needed to meet the learning criterion of 8 out of 10 correct decisions.

#### Experiment 3: Flexibility and re-learning

To test flexibility and re-learning we conducted a reversal experiment in which the bats had to learn to visit the box opposite the formerly rewarded one (fig. 2a). If a bat entered the formerly rewarded arm it was denied access to the mealworms by closing the entrance to the box. We recorded how many trials were required for a bat to investigate a new arm; either straight ahead or the opposite box; we used this metric as a measure of flexibility. After visiting the newly rewarded arm for the first time, an animal could learn where to find the reward. We then analyzed the number of days it took an animal from the first correct visit until making 8 of 10 trials correct in one session, and used this as a measure of re-learning. One *M. capaccinii* did not learn the new position within 15 sessions and was therefore excluded from the second part of experiment 3 as well as from experiment 4.

#### Experiment 4: Complex rule learning

For experiment 4, two additional arms were added to the maze (Figure 2b). Upon choosing the same arm as was rewarded in experiment 3 the bat now entered a second decision box and had to turn in the opposite direction as before (either left-right or right-left) to obtain the mealworm reward. We used the same criterion and analysis as in experiment 3 to quantify how quickly the bats learned this more complex task. Due to the length of the study (see above) and constraints on the timing and use of the field station, logistical reasons made it necessary to release two *M. emarginatus* before they could participate in this experiment.

Statistical analysis was conducted in R [49]. For the post-hoc analysis following a Kruskal-Wallis-test, we followed Dunn’s [50] suggestions for a test for multiple comparisons using rank sums.

## Results

### Experiment 1: Exploration

**Figure 3:**
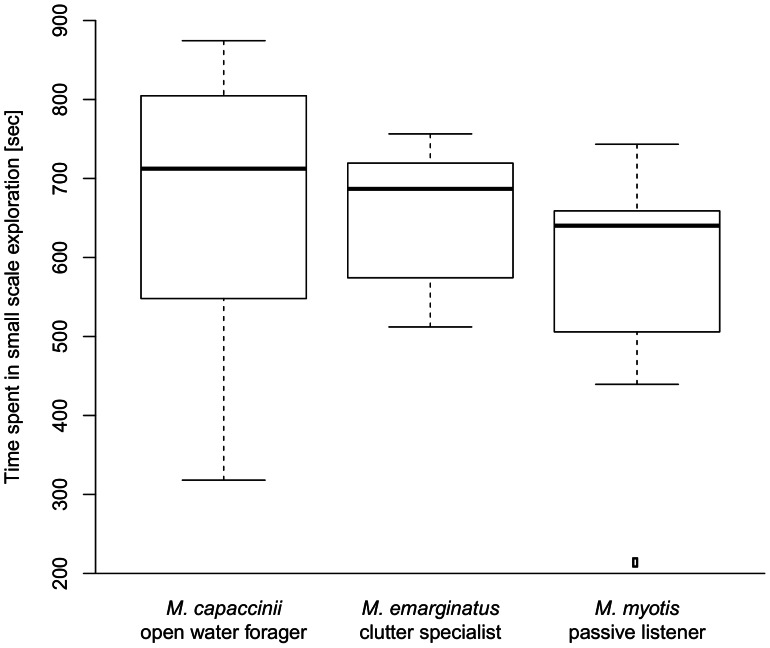
Experiment 1 (“Exploration”). Time spent with small scale exploration: There was no difference among the species in the time the bats spent with small scale exploration (p = 0.49). We found no difference in other parameters measured, except for the total number of mealworms eaten (not shown here).

For most behavioral parameters we did not find any differences between species in the exploration experiment. We compared the latency to exit the start box (F_2;17_ = 1.20; p = 0.325), the time spent with small scale exploration (F_2;17_ = 0.75; p = 0.489) (see fig. 3), the time spent walking fast (F_2;17_ = 0.06; p = 0.943), and the time spent immobile (F_2;17_ = 0.56; p = 0.581). All box-and-whisker-plots show median, 25 percentile, 75 percentile, minimum, and maximum. Outliers have values at least 1.5 times the interquartile range (IQR) larger than the 75 percentile or 1.5 times the IQR smaller than the 25 percentile. On average it took the bats less than one minute to leave the start box and begin to explore the maze (mean = 0.58 min ± SD = 0.80). They spent ten minutes on small scale exploration (10.37 min ±2.71), two minutes on fast walking (1.8 min ±1.34), and three minutes immobile (2.75 min ±2.94). Three *M. capaccinii*, four *M. emarginatus*, and five *M. myotis* visited all three boxes. There was no difference in the number of boxes visited among the species (Kruskal-Wallis: df = 2; chi-squared = 0.51; p = 0.776). We only found differences in the absolute number of mealworms consumed (Kruskal-Wallis: df = 2; chi-squared = 8.714; p = 0.013) with *M. myotis* eating the most and *M. capaccinii* eating the least. To control for the effect of body mass we calculated the number of mealworms eaten per gram body mass of the bat and still found a difference in the relative number of mealworms consumed (F_2;17_ = 7.73; p = 0.004) with *M. emarginatus* eating the most and *M. capaccinii* eating the least.

### Experiment 2: Simple Rule Learning

**Figure 4:**
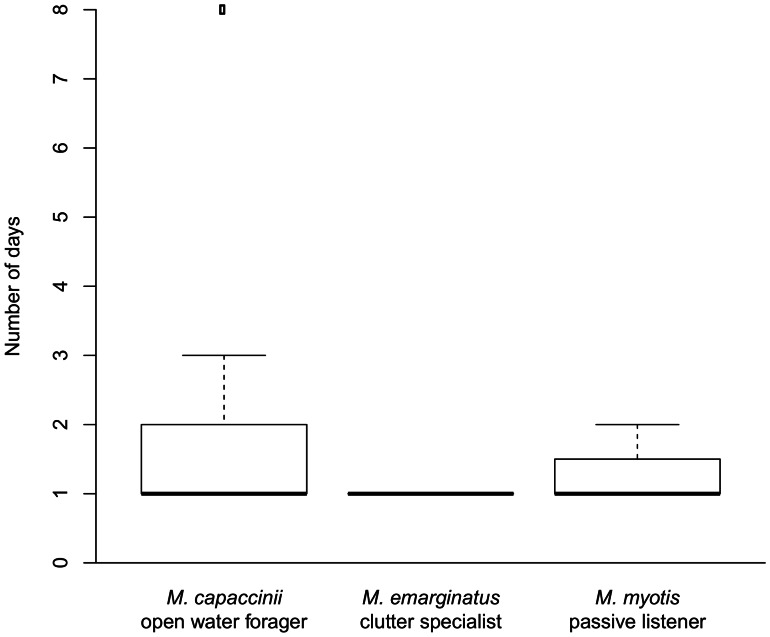
Experiment 2 (“Simple Rule Learning”). Number of days to reach criterion: There was no difference in learning performance among the three species (p = 0.34). Most animals reached the criterion on the first day after pre-training.

All species learned the task quickly (see fig. 4). After two days of training, there were no differences among any of the species in the number of days the bats needed to make 8 out of 10 correct decisions (Kruskal-Wallis: df = 2; chi-squared = 2.16; p = 0.340).

### Experiment 3: Flexibility and Re-learning

**Figure 5:**
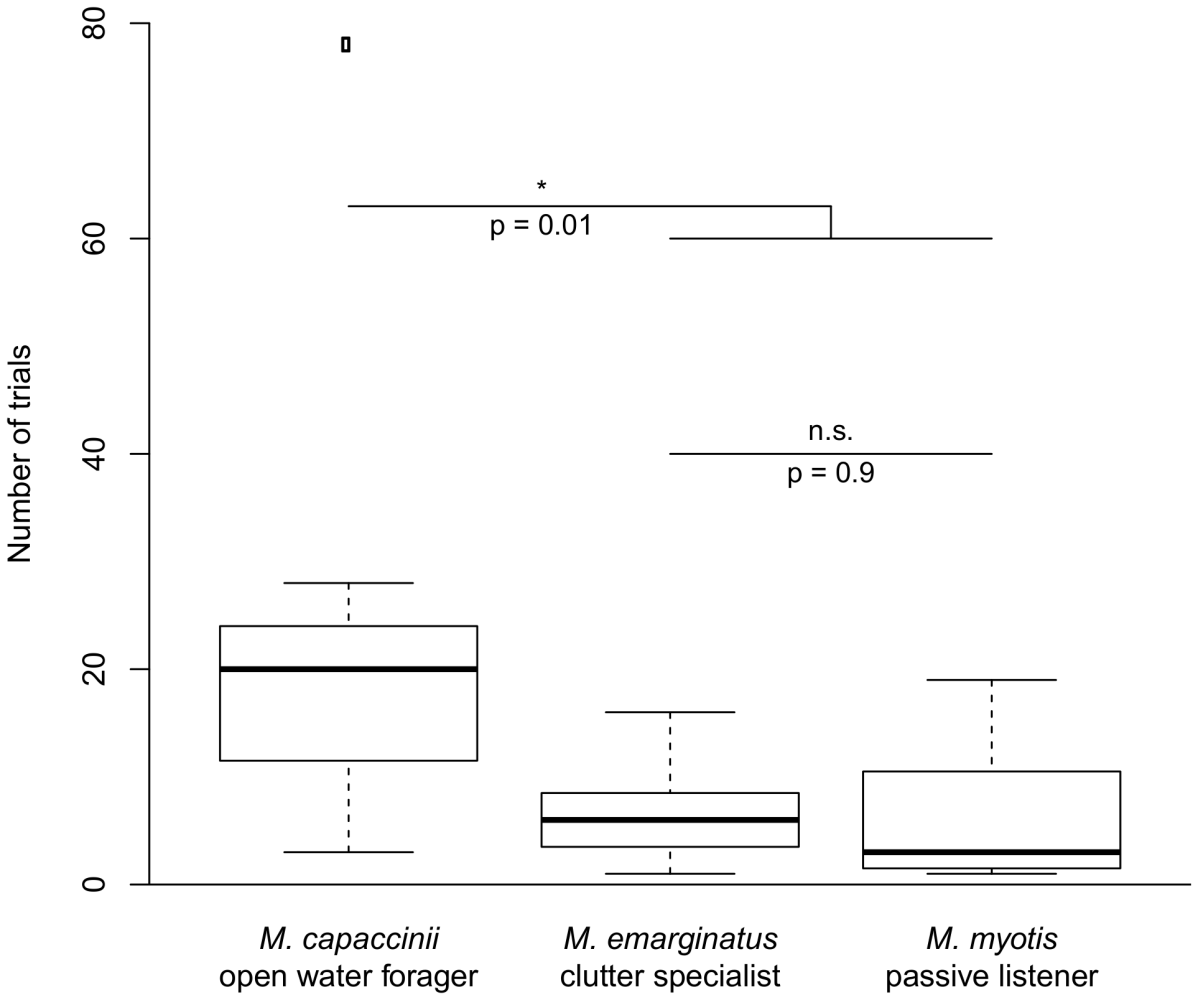
Experiment 3 (“Flexibility”). Number of trials until the animals visited a formerly unrewarded arm for the first time.

Most *M. emarginatus* and *M. myotis* visited a new arm on the first day (trial 1 to 10), while most *M. capaccinii* required at least two days to visit a new arm for the first time (fig. 5 and supporting information Video S1). A Kruskal-Wallis-test showed significant differences between the species (df = 2; chi-squared = 7.68; p = 0.022). One *M. capaccinii* was an extreme outlier that required 78 trials to try a different arm. To ensure that the difference between the species was not due to this single individual, we tentatively excluded it from analysis and still found significant differences among the species (Kruskal-Wallis: df = 2; chi-squared = 6.30; p = 0.04). We did a post-hoc analysis following Dunn [50] to find out if there were differences between *M. capaccinii* and the other two species, as well as differences between *M. emarginatus* and *M. myotis*. *M. capaccinii* required significantly more trials to change its strategy and search in a new location than did the two other species. There was no difference in the number of trials required for *M. emarginatus* and *M. myotis* (fig. 5 and supporting information Video S1).

**Figure 6:**
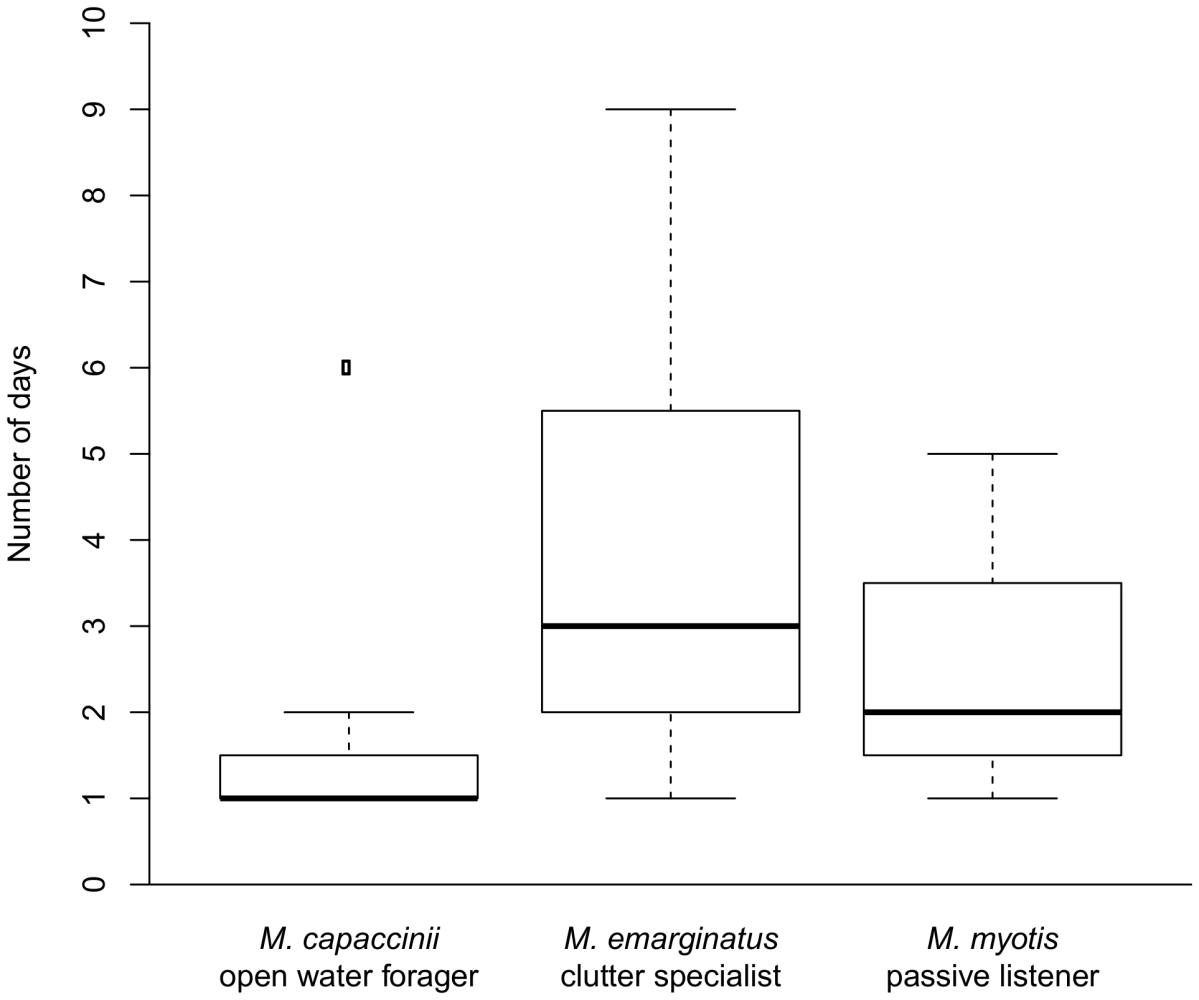
Experiment 3 (“Re-learning”). Number of days from visiting the newly rewarded box for the first time to reaching criterion (turning 8 out of 10 times in the opposite direction than before): There was no difference in learning performance among the species (p = 0.13).

We found no difference among species in re-learning speed (Kruskal-Wallis: df = 2; chi-squared = 4.14; p = 0.126, fig. 6). However, when the *M. capaccinii* outlier (which needed six days to achieve 8 of 10 correct trials) was removed from the analysis, we found a significant difference among the species (Kruskal-Wallis: df = 2; chi-squared = 6.91; p = 0.032), with a faster re-learning speed in *M. capaccinii* than in the other species (Dunn: p = 0.022).

### Experiment 4: Complex Learning

**Figure 7:**
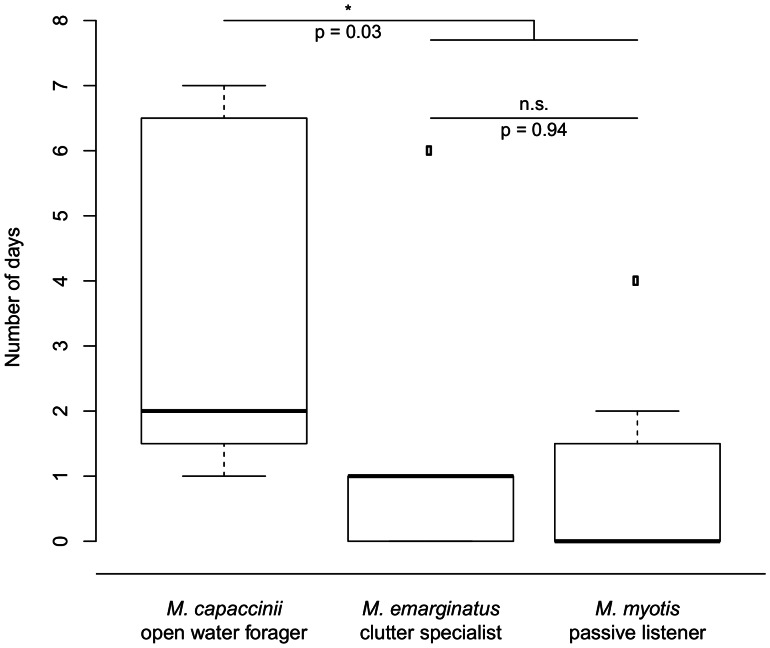
Experiment 4 (“Complex Rule Learning”). Number of days from visiting the newly rewarded box for the first time to reaching learning criterion.

A Kruskal-Wallis-test showed a borderline significant difference among all species (df = 2; chi-squared = 5.81; p = 0.055) in the number of days needed to make 8 out of 10 correct choices. In the post-hoc analysis we conducted following Dunn [50] we found a clearly significant difference between *M. capaccinii* and the other two species (p = 0.03), implying that it took *M. capaccinii* longer to learn this more complex task of turning twice than it took either of the other species. The two *M. myotis* and *M. emarginatus* outliers (fig. 7) coupled with a low overall sample size (7 *M. capaccinii*, 5 *M. emarginatus*, 7 *M. myotis*) lead to only borderline significance in the Kruskal-Wallis-test (Kruskal-Wallis without outliers: df = 2; chi-squared = 8.65; p = 0.013). Again, there was no difference between *M. emarginatus* and *M. myotis* (fig. 7).

For the experiments 2–4 we investigated whether there were differences among the individuals that were first trained to turn right versus the ones that were trained to the left box. We found no differences for any parameter between these two groups (all p>0.1).

## Discussion

We find strong evidence that bat cognitive skills reflect their ecological niche. By comparing the ability to find food in a species-fair, artificial crawling maze we tested the learning performance of three closely related species that naturally forage in habitats differing in complexity, stability, and food predictability. We found that bat species hunting in more complex, less stable habitats with lower food predictability perform better in more complex learning tasks and are more flexible when the food source is relocated than those hunting in simple, more stable habitats with highly predictable food resources. Our results confirm the “Environmental Complexity Thesis” [10] and are the first to directly link bat learning and flexibility with ecological foraging niche.

### Learning in an Artificial Environment

The comparison of learning performance of different animal species is extremely challenging [51]. To allow for comparison all species should be tested in the same setup, while each specieś unique set of sensorial and motor skills has to be accounted for to guarantee species-fairness. While a less artificial, more natural foraging task would have offered greater insight into the cognitive capabilities of these animals in nature, natural foraging tasks would have hindered cross-species comparisons. To compare learning and flexibility of taxa with widely disparate foraging ecologies (the goal of this study), we purposefully chose experimental tasks that were foreign to all. We used the exploration phase to assess possible differences in motor skills among the three species that might have hindered access and mobility within the maze. The lack of differences among the investigated species in the exploration experiment suggests that our results were not biased by any consistent species differences in the ability to cope with the maze. The difference in the number of mealworms eaten during exploration could be explained by body size differences or slight sensory inequalities. In the other experiments there were more mealworms in the boxes and the animals were given all the time they needed to consume two to four mealworms before the next trial started. This should have compensated for possible differences in detection and eating speed. We therefore conclude that the results of our experiments were not affected by differences in size or crawling performance among the species and that all animals had equal opportunities to learn about all the boxes and paths of the maze. The plastic maze was equally artificial for all bats and should not have posed a particular disadvantage to any one species. Because of its artificial novelty, it proved effective in elucidating consistent species differences in learning performance. Our results demonstrate that an artificial setup like ours can be useful to balance potentially biasing factors and to investigate cognitive abilities of different species in a species-fair manner.

### Solving Simple and Complex Tasks

Two days of pre-training were sufficient to teach the bats to find food in one arm of the maze. These results show that the process of learning a simple rule (experiment 2) is quick, and suggests that simple rule learning is not sufficiently challenging to elicit differential responses in the species tested. It suggests that differences in cognitive abilities might only be detectable in more complex tasks.

Indeed, when the bats were required to exhibit more complex behavior to obtain a food reward – to turn twice to find the rewarded box in the complex learning task (experiment 4) – we found differences among the species. *M. myotis* easily completed this task, and most individuals met the learning criterion on the first day of testing. *M. emarginatus* also quickly learned to find and remember the new feeding box, most individuals learning within days 1–2. *M. capaccinii* showed the greatest inter-individual variation. Of the seven *M. capaccinii*, four learned the task rather quickly within days 1–2 and three learned the task very slowly, requiring six to seven days. Intermediate learning performance was not observed in this species. Interestingly, the one *M. capaccinii* that required 78 trials to investigate a new arm in experiment 3 (flexibility and re-learning) was the fastest learner in this task and succeeded in finding the food reward in 8 out of 10 trials during the first night. Thus, the least flexible individual was the fastest learner in the complex learning task. Even though the sample size in the complex rule learning experiment was smaller than in the other experiments and the results only show borderline significance (p = 0.055), this trend suggests that *M. capaccinii* needed the greatest number of days to remember the newly rewarded box. A larger dataset would be needed to demonstrate clearly significant results. However, while *M. capaccinii* were able to learn a simple rule as quickly as the other species, a more complicated path was more difficult for them to learn and remember. Thus, as predicted, the species foraging in the least complex environment, open water, performed more poorly than the passive listening gleaner and the clutter specialist.

In experiment 4, a few individuals of each of the three species went straight through the center box several times, even though the end box of this arm had never been rewarded. This behavior rarely occurred in the other experiments. It would be interesting to investigate if the bats had developed a cognitive map of the maze and would have turned in the direction of the rewarded box at the end of the arm or would have been able to perform shortcuts or use a more direct route, had this been available. Further investigations are necessary to determine whether insectivorous bats use cognitive maps in foraging as has been recently shown for fruit-eating bats on a larger scale [52], and if so, the conditions under which they do so.

### Flexibility and Re-learning

As expected, we found a clear difference among the three species in the flexibility test (experiment 3). While most *M. emarginatus* and *M. myotis* tried new ways to find food on the first day when the familiar route was blocked, the open water forager, *M. capaccinii*, was persistent in visiting the formerly rewarded box. This supports the “Environmental Complexity Thesis” [10], which predicts that bats hunting in more complex and unpredictable habitats (for example, in or near vegetation) will show more flexible behavior than bats hunting in less complex and more predictable habitats (for example, over water surfaces). Game theoretical modeling suggests that individuals can enhance their probability of finding food by specializing on one food type when the location of their food sources is predictable in space and time [1]. The stereotypic behavior of *M. capaccinii* together with what we know from natural history and dietary studies [30], [31] suggests that rather than specializing on a single food type, *M. capaccinii* has evolved to specialize on a specific and simple foraging habitat in which its particular foraging skills excel. The obstinacy of this species suggests that stereotypic behavior under natural conditions is beneficial and could be genetically determined. It is interesting that once the bats discovered the new food source in our artificial maze, there was no difference in the re-learning speed of a simple rule (e.g., always turn right or always turn left). There even was a trend that *M. capaccinii* learned the new position faster than the other species. This might be due to their greater persistence. This trend suggests that persistence can have evolutionary benefits. It is possible that in nature *M. emarginatus* and *M. myotis* continuously sample their surroundings for more and (potentially) better food sources, thereby making mistakes, while *M. capaccinii* persists in repeatedly visiting locations with a high probability of containing food. It is interesting to note that there is evidence that *M. capaccinii* in Israel have recently begun hunting fish adding this prey to their diet only within the last century. Especially during winter they forage to a great extent on *Gambusia affinis*, a species that was introduced in the area in the 1920s [53]. Occasional piscivory is known in this species from other areas and seems to be a common foraging strategy in other trawling bat species in times of high fish abundance [31], [54], [55]. In an experimental setup with *M. capaccinii* in the flight cage, dips into the water were not directionally targeting fish, but were carried out at random and seemed to follow stereotyped patterns [54], similar perhaps to the stereotyped foraging behavior we found in *M. capaccinii* in our maze experiment.

One *M. capaccinii* could not be included in the full set of experiments because it failed to complete the re-learning task within 15 sessions (experiment 3). This individual visited a new arm in the second night of experiment 3 (trial 20) and found the new food source for the first time in the fifth night of experiment 3 (trial 42), but even after ten additional nights of testing, never learned to consistently visit the newly rewarded box. This particular individual also required the most time of all bats to complete the simple rule learning task (see fig. 4; one *M. capaccinii* requiring eight days). This behavior differs from the other *M. capaccinii* which showed learning and re-learning within one or two nights once they found the food source, and highlights the potential for high inter-individual variation even within a species. Apart from this individual we did not find that any other bat consistently showed a different performance from its conspecifics (i.e. the outliers in different experiments were different individuals).

The relationship between flexibility and stereotypy and food predictability has been studied on an intraspecific level in the context of animal personality research. Studies of intraspecific variation in exploration speed, flexibility, and novelty response in birds [56] and small mammals [57] show that individuals that are more persistent and do not change their behavior when presented with changes in food positioning site have the advantage of finding more food under stable conditions. On the other hand, once they find a reliable food source they develop inflexible, routine-based behavior. Therefore, under more variable conditions, they have a disadvantage compared to less persistent individuals. Depending on how variable a specific environment of an individual or a population is, we predict a selective pressure to shape foraging behavior either towards more stereotypy or towards more flexibility. Because in our maze experiments we see predictable differences across species despite favorable conditions to the contrary, we further predict a genetic basis for stereotyped versus more flexible behavior, similar to the genetic basis for stress and novelty response [58], exploration propensity [59], and aggressive behavior [57]. It is possible that within-species differences on the flexibility-stereotypy gradient ultimately translate into species differences in these traits, as shown in the present study, through allopatric or even sympatric speciation with differential microhabitat selection.

### Brain-size and Cognitive Abilities

Ratcliffe et al. [60] categorized predatory bat species into groups based on their natural foraging modes. They found that bat species they categorized as more flexible in their hunting strategies and ground gleaning species like *M. myotis* have larger relative brain sizes and a larger neocortex than less flexible species, such as open space aerial hawking bats. Eisenberg and Wilson [61] also found that aerial insectivores have smaller relative brain sizes than other insectivores, which in turn have smaller relative brain sizes than frugivorous bats. Several studies in birds and mammals show that the relative size of the neocortex as well as the relative size of the whole brain are strongly related to cognitive abilities and enhanced novelty response (e.g. [62], [63] also reviewed in [64]). We thus predicted that *M. emarginatus* and *M. myotis* should have larger relative brains and neocortices than *M. capaccinii*. As data on brain sizes of *M. emarginatus* and *M. capaccinii* are not available yet, this prediction remains to be studied. However, available data on skull morphology indicate that *M. capaccinii* indeed has the smallest relative skull and hence potentially the smallest relative brain of our three species followed by *M. emarginatus* and then *M. myotis* (condylobasal length divided by forearm length and zygomatic width divided by forearm length; data from [65]).

### Ecology and Sociality

Animal cognition researchers generally attribute the evolution of brains and intelligence to either ecology (e.g. [2]) or to social structure (e.g. [66]). Recently, however, there are models that combine the two (e.g. [1]). In our case, we have chosen bat species that differ widely in ecology, but are quite similar in their social systems. In all three species, females congregate in large maternity groups of up to several thousand individuals while males roost singly or in smaller bachelor colonies in summer; in winter, males and females from all three species hibernate singly or in small clusters ([67] *M. capaccinii* p. 213; *M. emarginatus* p. 244; *M. myotis* p. 254). In some cases pregnant or lactating females of all three species can be found in the same cave at the same time in Bulgaria (TC, personal observation). By choosing species with very similar social structures and other aspects of their biology, but disparate foraging ecologies, we can infer that the differences we find in learning and flexibility reflect ecology and not sociality.

### Conclusion

Results from this study confirm our prediction that open water foragers are more stereotyped and hence less flexible than species hunting in more complex habitats. Contrary to our expectations, there was a trend that open water foragers learn simple rules more quickly than clutter specialists and passive listening gleaners. Given a more complex task involving two decisions, however, passive listening gleaners and clutter specialists showed a tendency to outperform bats hunting over water. Due to their unstructured foraging habitat, we would expect results from bats hunting in open space to be similar to those of open water hunters. More subtle differences between passive listening gleaners and clutter specialists might only be revealed in a yet more complex task.

Our data support the hypothesis that cognitive abilities of animals are shaped by the demands of their ecological background. Our results concur with those of recent studies on nectar-feeding bats that differ in their spatial working memory performance depending on their degree of dietary specialization, in which a specialized nectarivorous bat foraged more efficiently at artificial flower patches than a generalist that also includes fruits and insects in its diet [68]. They are also in line with recent findings for birds (e.g. [5], [6]) and mammals [9].

## Supporting Information

Video S1 **Video clips demonstrate** ***M. capaccinii*** **navigating the maze in the flexibility and re-learning task and in the complex learning task.** For comparisons of crawling performance, video clips demonstrate *M. myotis*, a much larger species, in the flexibility and re-learning task.
(WMV)

## References

[1] Overington SE, Dubois F, Lefebvre L (2008) Food unpredictability drives both generalism and social foraging: a game theoretical model. Behavioral Ecology 19: 836–841.

[2] Parker S, Gibson K (1977) Object manipulation, tool use and sensorimotor intelligence as feeding adaptations in Cebus monkeys and great apes. Journal of Human Evolution 6: 623–641.

[3] Rozin P, Kalat JW (1971) Specific hungers and poison avoidance as adaptive specializations of learning. Psychological Review 78: 459–486. 4941414 10.1037/h0031878

[4] Dukas R, Ratcliffe JM (2009) Introduction. In: Dukas R, Ratcliffe JM, editors. Cognitive Ecology II. Chicago: University Of Chicago Press.

[5] Mettke-Hofmann C, Gwinner E (2003) Long-term memory for a life on the move. Proceedings of the National Academy of Sciences of the United States of America 100: 5863–5866. 12719527 10.1073/pnas.1037505100 PMC156292

[6] Gibson BM, Kamil AC (2005) The fine-grained spatial abilities of three seed-caching corvids. Learning & behavior 33: 59–66. 15971493 10.3758/bf03196050

[7] Micheli F (1997) Effects of experience on crab foraging in a mobile and a sedentary species. Animal behaviour 53: 1149–1159. 9236012 10.1006/anbe.1996.0349

[8] Day L, Crews D, Wilczynski W (1999) Spatial and reversal learning in congeneric lizards with different foraging strategies. Animal behaviour 57: 393–407. 10049480 10.1006/anbe.1998.1007

[9] Haupt M, Eccard J, Winter Y (2010) Does spatial learning ability of common voles (*Microtus arvalis*) and bank voles (*Myodes glareolus*) constrain foraging efficiency? Animal cognition 13: 783–791. 20596739 10.1007/s10071-010-0327-8

[10] Godfrey-Smith P (2001) Environmental complexity and the evolution of cognition. In: Sternberg R, Kaufman J, editors. The evolution of intelligence. Mahwah, New Jersey: Lawrence Erlbaum Associates London. pp.223–250.

[11] Tebbich S, Stankewitz S, Teschke I (2012) The relationship between foraging, learning abilities and neophobia in two species of Darwin’s finches. Ethology 118: 135–146.

[12] Simmons NB (2005) Order Chiroptera. In: Wilson DE, Reeder DM, editors. Mammal species of the world: a taxonomic and geographic reference, vol 1. Baltimore: John Hopkins University Press. pp.312–529.

[13] Simmons NB (2005) An Eocene big bang for bats. Science 307: 527–528. 15681371 10.1126/science.1108871

[14] Norberg UM, Rayner JM (1987) Ecolocigal morphology and flight in bats (Mammalia; Chiroptera): wing adaptations, flight performance, foraging strategy and echolocation. Philosophical Transactions of the Royal Society B 316: 335–427.

[15] Jones G, Holderied MW (2007) Bat echolocation calls: adaptation and convergent evolution. Proceedings of the Royal Society B 274: 905–912. 17251105 10.1098/rspb.2006.0200 PMC1919403

[16] Jones G, Teeling EC (2006) The evolution of echolocation in bats. Trends in Ecology & Evolution 21: 149–156. 16701491 10.1016/j.tree.2006.01.001

[17] Schnitzler H-U, Moss C, Denzinger A (2003) From spatial orientation to food acquisition in echolocating bats. Trends in Ecology & Evolution 18: 386–394.

[18] Weinbeer M, Kalko EKV (2007) Ecological niche and phylogeny: the highly complex echolocation behavior of the trawling long-legged bat, *Macrophyllum macrophyllum* . Behavioral Ecology and Sociobiology 61: 1337–1348.

[19] Meyer CFJ, Weinbeer M, Kalko EKV (2005) Home-range size and spacing patterns of *Macrophyllum macrophyllum* (Phyllostomidae) foraging over water. Journal of Mammalogy 86: 587–598.

[20] Kalko EKV, Schnitzler H-U (1989) The echolocation and hunting behavior of Daubenton’s bat, *Myotis daubentoni* . Behavioral Ecology and Sociobiology 24: 225–238.

[21] Schnitzler H-U, Kalko EKV, Kaipf I, Grinnell AD (1994) Fishing and echolocation behavior of the greater bulldog bat, *Noctilio leporinus*, in the field. Behavioral Ecology and Sociobiology 35: 327–345.

[22] Siemers BM (2001) Finding prey by associative learning in gleaning bats: experiments with a Nattereŕs bat *Myotis nattereri* . Acta Chiropterologica 3: 211–215.

[23] Safi K, Dechmann DKN (2005) Adaptation of brain regions to habitat complexity: a comparative analysis in bats (Chiroptera). Proceedings of the Royal Society B 272: 179–186. 15695209 10.1098/rspb.2004.2924 PMC1634959

[24] Boonman AM, Boonman M, Bretschneider F, Van de Grind WA (1998) Prey detection in trawling insectivorous bats: duckweed affects hunting behaviour in Daubenton’s bat, *Myotis daubentonii* . Behavioral Ecology and Sociobiology 44: 99–107.

[25] Siemers BM, Stilz P, Schnitzler H-U (2001) The acoustic advantage of hunting at low heights above water: behavioural experiments on the European “trawling” bats *Myotis capaccinii*, *M. dasycneme* and *M. daubentonii* . The Journal of experimental biology 204: 3843–3854. 11807102 10.1242/jeb.204.22.3843

[26] Siemers BM, Baur E, Schnitzler H-U (2005) Acoustic mirror effect increases prey detection distance in trawling bats. Die Naturwissenschaften 92: 272–276. 15871000 10.1007/s00114-005-0622-4

[27] Greif S, Siemers BM (2010) Innate recognition of water bodies in echolocating bats. Nature communications 1: DOI: 10.1038/ncomms1110. doi:10.1038/ncomms1110. 10.1038/ncomms1110 PMC3060641 21045825

[28] Ciechanowski M, Zając T, Zielińska A, Dunajski R (2010) Seasonal activity patterns of seven vespertilionid bat species in Polish lowlands. Acta Theriologica 55: 301–314.

[29] Ciechanowski M, Zając T, Biłas A, Dunajski R (2007) Spatiotemporal variation in activity of bat species differing in hunting tactics: effects of weather, moonlight, food abundance, and structural clutter. Canadian Journal of Zoology 85: 1249–1263.

[30] Almenar D, Aihartza J, Goiti U, Salsamendi E, Garin I (2006) Habitat selection and spatial use by the trawling bat *Myotis capaccinii* (Bonaparte, 1837). Acta Chiropterologica 8: 157–167.

[31] Biscardi S, Russo D, Casciani V, Cesarini D, Mei M, et al (2007) Foraging requirements of the endangered long-fingered bat: the influence of micro-habitat structure, water quality and prey type. Journal of Zoology 273: 372–381.

[32] Jones PL, Page RA, Hartbauer M, Siemers BM (2010) Behavioral evidence for eavesdropping on prey song in two Palearctic sibling bat species. Behavioral Ecology and Sociobiology 65: 333–340.

[33] Arlettaz R (1996) Feeding behaviour and foraging strategy of free-living mouse-eared bats, *Myotis myotis* and *Myotis blythii* . Animal Behaviour 51: 1–11.

[34] Goerlitz HR, Siemers BM (2007) Sensory ecology of prey rustling sounds: acoustical features and their classification by wild grey mouse lemurs. Functional Ecology 21: 143–153.

[35] Arlettaz R, Perrin N, Hausser J (1997) Trophic resource partitioning and competition between the two sibling bat species *Myotis myotis* and *Myotis blythii* . Journal of animal ecology 66: 897–911.

[36] Russo D, Jones G, Arlettaz R (2007) Echolocation and passive listening by foraging mouse-eared bats *Myotis myotis* and *M. blythii* . The Journal of Experimental Biology 210: 166–176. 17170159 10.1242/jeb.02644

[37] Siemers BM, Güttinger R (2006) Prey conspicuousness can explain apparent prey selectivity. Current biology 16: R157–R159. 16527730 10.1016/j.cub.2006.02.056

[38] Schnitzler H-U, Kalko EKV (2001) Echolocation by insect-eating bats. BioScience 51: 557–569.

[39] Siemers BM, Schnitzler H-U (2004) Echolocation signals reflect niche differentiation in five sympatric congeneric bat species. Nature 429: 657–661. 15190352 10.1038/nature02547

[40] Wang J, Kanwal J, Zhang C, Jiang T, Lu G, et al (2010) Seasonal habitat use by greater horseshoe bat *Rhinolophus ferrumequinum* (Chiroptera: Rhinolophidae) in Changbai Mountain temperate forest, Northeast China. Mammalia 74: 257–266.

[41] Flaquer C, Puig-Montserrat X, Burgas A, Russo D (2008) Habitat selection by Geoffroýs bats (*Myotis emarginatus*) in a rural Mediterranean landscape: implications for conservation. Acta Chiropterologica 10: 61–67.

[42] Krull D, Schumm A, Metzner W, Neuweiler G (1991) Foraging areas and foraging behavior in the notch-eared bat, *Myotis emarginatus* (Vespertilionidae). Behavioral Ecology and Sociobiology 28: 247–253.

[43] Schumm A, Krull D, Neuweiler G (1991) Echolocation in the notch-eared bat, *Myotis emarginatus* . Behavioral Ecology and Sociobiology 28: 255–261.

[44] Zahn A, Bauer S, Kriner E, Holzhaider J (2009) Foraging habitats of *Myotis emarginatus* in Central Europe. European Journal of Wildlife Research 56: 395–400.

[45] Stadelmann B, Lin L-K, Kunz TH, Ruedi M (2007) Molecular phylogeny of New World Myotis (Chiroptera, Vespertilionidae) inferred from mitochondrial and nuclear DNA genes. Molecular phylogenetics and evolution 43: 32–48. 17049280 10.1016/j.ympev.2006.06.019

[46] Page RA, Von Merten S, Siemers BM (2012) Associative memory or algorithmic search: A comparative study on learning strategies of bats and shrews. Animal cognition 15: 495–504. 22391618 10.1007/s10071-012-0474-1

[47] Ruczynski I, Siemers BM (2011) Hibernation does not affect memory retention in bats. Biology letters 7: 153–155. 20702450 10.1098/rsbl.2010.0585 PMC3030893

[48] Canadian Council On Animal Care (2003) CCAC species-specific recommendations on: BATS: 1–9. Available: http://www.ccac.ca/Documents/Standards/Guidelines/Add_PDFs/Wildlife_Bats.pdf. Accessed 2013 April 24.

[49] R Development Core Team (2012) R: A language and environment for statistical computing. Available: http://www.r-project.org/Accessed 2013 April 24.

[50] Dunn OJ (1964) Multiple comparisons using rank sums. Technometrics 6: 241–252.

[51] Bitterman ME (1975) The comparative analysis of learning - are the laws of learning the same in all animals? Science 188: 699–709. 17755167 10.1126/science.188.4189.699

[52] Tsoar A, Nathan R, Bartan Y, Vyssotski A, Dell’Omo G, et al (2011) Large-scale navigational map in a mammal. Proceedings of the National Academy of Sciences of the United States of America 108: 718–724. 10.1073/pnas.1107365108 PMC3174628 21844350

[53] Levin E, Barnea A, Yovel Y, Yom-Tov Y (2006) Have introduced fish initiated piscivory among the long-fingered bat? Mammalian Biology - Zeitschrift für Säugetierkunde 71: 139–143.

[54] Aihartza J, Almenar D, Salsamendi E, Goiti U, Garin I (2008) Fishing behaviour in the long-fingered bat *Myotis capaccinii* (Bonaparte, 1837): an experimental approach. Acta Chiropterologica 10: 287–301.

[55] Aihartza J, Goiti U, Alemar D, Garin I (2003) Evidences of piscivory by *Myotis capaccinii* (Bonaparte, 1837) in southern Iberian Peninsula. Acta chiropterologica 5: 193–198.

[56] Verbeek M, Drent P, Wiepkema P (1994) Consistent individual differences in early exploratory behaviour of male great tits. Animal Behaviour 48: 1113–1121.

[57] Benus R, Bohus B, Koolhaas J, Van Oortmerssen GA (1991) Heritable variation for aggression as a reflection of individual coping strategies. Experientia 47: 1008–1019. 1936199 10.1007/BF01923336

[58] Ruiz-Gomez MDL, Huntingford FA, Øverli Ø, Thörnqvist P-O, Höglund E (2011) Response to environmental change in rainbow trout selected for divergent stress coping styles. Physiology & behavior 102: 317–322. 21130105 10.1016/j.physbeh.2010.11.023

[59] Dingemanse NJ, De Goede P (2004) The relation between dominance and exploratory behavior is context-dependent in wild great tits. Behavioral Ecology 15: 1023–1030.

[60] Ratcliffe JM, Fenton MB, Shettleworth SJ (2006) Behavioral flexibility positively correlated with relative brain volume in predatory bats. Brain, behavior and evolution 67: 165–176. 10.1159/000090980 16415571

[61] Eisenberg J, Wilson D (1978) Relative brain size and feeding strategies in the Chiroptera. Evolution 32: 740–751. 28567922 10.1111/j.1558-5646.1978.tb04627.x

[62] Reader SM, Laland KN (2002) Social intelligence, innovation, and enhanced brain size in primates. Proceedings of the National Academy of Sciences of the United States of America 99: 4436–4441. 11891325 10.1073/pnas.062041299 PMC123666

[63] Sol D, Duncan RP, Blackburn TM, Cassey P, Lefebvre L (2005) Big brains, enhanced cognition, and response of birds to novel environments. Proceedings of the National Academy of Sciences of the United States of America 102: 5460–5465. 15784743 10.1073/pnas.0408145102 PMC556234

[64] Lefebvre L, Sol D (2008) Brains,lifestyles and cognition: are there general trends? Brain, behavior and evolution 72: 135–144. 10.1159/000151473 18836259

[65] Krapp F, Niethammer J, editors (2011) Die Fledermäuse Europas. 1st ed. Wiebelsheim: AULA-Verlag GmbH.

[66] Byrne RW, Bates LA (2007) Sociality, evolution and cognition. Current biology 17: R714–723. 17714665 10.1016/j.cub.2007.05.069

[67] Dietz C, Von Helversen O, Nill D (2009) Bats of Britain, Europe and Northwest Africa. 1st ed. London: A & C Black Publishers Ltd.

[68] Henry M, Stoner KE (2011) Relationship between spatial working memory performance and diet specialization in two sympatric nectar bats. PLoS ONE 6: e23773 doi:10.1371/journal.pone.0023773 21931612 10.1371/journal.pone.0023773 PMC3170290

